# Therapeutic efficacy of equine botulism antitoxin in Rhesus macaques

**DOI:** 10.1371/journal.pone.0186892

**Published:** 2017-11-22

**Authors:** Shantha Kodihalli, Andrew Emanuel, Teresa Takla, Yi Hua, Charles Hobbs, Ross LeClaire, Denise C. O’Donnell

**Affiliations:** 1 Research and Development, Emergent BioSolution, Winnipeg, Manitoba, Canada; 2 Clinical Research, Emergent BioSolutions, Winnipeg, Manitoba, Canada; 3 Lovelace Respiratory Research Institute, Albuquerque, New Mexico, United States of America; Institut Pasteur, FRANCE

## Abstract

**Background:**

There are currently no licensed vaccines available for prevention of botulism in humans. The vaccination is not desirable due to expanding therapeutic indications of botulinum toxins. The only available specific treatment for botulism is antitoxin to remove circulating toxin, thus, preventing further neuronal damage. BAT^®^ (Botulism Antitoxin Heptavalent (A, B, C, D, E, F, G)—(Equine)) has been developed and its therapeutic efficacy evaluated against botulinum neurotoxin serotype A (BoNT/A) in Rhesus macaques.

**Methods and findings:**

In a post-exposure prophylaxis (PEP) study, animals were exposed to 4x LD_50_/kg of BoNT/A and administered intravenously with either BAT (1x or 0.1x scaled human dose), or placebo at 4 hours post-exposure. The animals were monitored for 14 days. For the therapeutic intervention studies, animals were exposed to a 1.7x LD_50_/kg of BoNT/A and treated intravenously with either placebo or BAT at a 1x scaled human dose at the onset of clinical signs. Animals were monitored on an hourly basis for 14 or 21 days. In the PEP study, all animals tolerated equine based antitoxin without any adverse clinical signs. A 100% survival was observed in groups treated with the BAT compared to 0% survival in those treated with the placebo (p<0.001, Fisher’s exact test). BAT antitoxin prevented the development of signs of neurotoxicity of botulinum toxin. In a therapeutic study, treatment with the BAT at scaled 1x human dose after the onset of clinical signs significantly enhanced survival compared to the placebo (46.6% vs. 0%, p<0.0001, Fisher’s exact test). Additionally, treatment with the BAT delayed the progression of signs (muscular weakness, respiratory distress, oral/nasal discharge) of toxin intoxication and reduced the severity of the disease.

**Conclusions:**

A single dose of BAT, when administered to symptomatic monkeys, resulted in a statistically significant survival benefit compared to the placebo. Additionally, BAT completely protected monkeys from the clinical signs of intoxication and subsequent death when administered as PEP treatment. These data in part supported the licensure of BAT under the Animal Rule in the United States by the Food and Drug Administration.

## Introduction

Botulism is a rare paralytic illness caused by intoxication with one or more of the seven neurotoxins produced by bacterial organisms of the genus *Clostridium*. Botulinum toxin exists in seven antigenically distinct serotypes designated by the letters A through G [1]. A new strain of *Clostridium botulinum* producing type ‘H’ botulinum toxin has been reported recently [2], however, the toxin’s characterization studies indicate a chimeric BoNT/FA which can be neutralized by antitoxin A. The neutralization data supports classification of this toxin as a chimeric BoNT/FA toxin rather than a new serotype [3, 4].

Botulinum toxins block the release of neurotransmitter acetylcholine from synaptic vesicles at the neuromuscular junction of cholinergic nerve endings. This blockade of neurotransmitter release accounts for the flaccid paralysis and autonomic dysfunction that are characteristic of the disease botulism [5]. Botulinum neurotoxins (BoNTs) are considered some of the most potent toxins known to mankind and due to their extreme toxicity have become potential bio-warfare agents [6, 7, 8, 1]. The two most likely forms of botulism that could arise from the deliberate release of BoNT are foodborne and inhalational botulism [1, 9]. Infant botulism is the most common form of human botulism in the United States and results from intestinal toxemia due to the colonization of *Clostridium botulinum*, *Clostridium baratti*, *or Clostridium butyricum* in the lumen of the large intestine of infants younger than 1 year of age. Intravenous botulism immunoglobulin (BabyBIG^®^) was developed as a specific treatment for infant botulism in 2003 [10, 11]. Wound botulism is a relatively rare form of botulism diagnosed among intravenous drug users due to contaminated needles or impure heroin [12, 13, 14, 15, 16]. In all forms of botulism, regardless of route of exposure, the botulinum neurotoxins share the same unique multi-step mode of action, which includes binding, internalization, membrane translocation, intracellular traffic, and proteolytic degradation of the target [17]. Once toxin enters the body, it undergoes a short distribution and a long elimination phase. During the distribution phase, botulinum toxin migrates to the vicinity of susceptible cells, such as cholinergic nerve endings. Only these cells have the ability to selectively accumulate the molecule [18].

While an average of 145 botulism cases occur annually in the United States (US), botulism can pose a greater risk to the public. In 2014, a total of 161 cases of botulism were reported [19]. It is estimated that a point source aerosol release of BoNT in civilian population can incapacitate or kill 10% of the population within 0.5 km from the source [1]. Human botulism mortality rates have been reported as high as 60% [20,21], however, with the improved supportive care that includes respiratory support, antibiotics, and antitoxins, mortality rates have decreased significantly in recent years [22]. The duration of hospitalization and length of stay in intensive care units (ICUs) still continue to present a major burden to the healthcare system.

Humans are susceptible to all seven serotypes [23, 24]; however, serotypes A, B, and E are the most common. Serotype A has been the most prevalent serotype found in the United States [25] and is known to have the most sustained action which can vary from many weeks to many months [26, 27, 28]. Due to the severity of the clinical course caused by this serotype, a majority of the patients affected by BoNT/A require respiratory support [29]. There are currently no licensed vaccines available for prevention of botulism in humans.

Mass vaccinations for botulism are less desirable due to expanding therapeutic indications for BoNTs. The immune response induced by BoNT toxins render patients unresponsive to BoNT treatments. Progress in developing botulism therapies or preventive vaccines has been much slower, although the monoclonal antibody therapies have shown promise against some of the serotypes [30, 31]. A European consortium, successfully developed human-like neutralizing antibodies directed against BoNT/A, B, and E [32, 33, 34]. As there were no therapeutic options available to cover all seven botulinum serotypes, BAT was developed as a heptavalent drug product for the treatment of symptomatic botulism caused by BoNT serotypes A, B, C, D, E, F, and G in adults and pediatric patients. BAT is manufactured by combining equine plasma containing a single antitoxin serotype (A, B, C, D, E, F, or G) into the final heptavalent product. Although studies conducted in humans that received equine antitoxin has provided the basis for approval of equine antitoxin in the past, the incidence of human cases is too low to conduct carefully controlled clinical trials. Therefore, BAT was developed and evaluated for licensure in the US under 21 CFR Part 601 (Subpart H, Animal Rule), “Approval of Biological Products When Human Efficacy Studies Are Not Ethical or Feasible”. Under this rule, the approval is based on adequate and well-controlled animal efficacy studies to establish that the drug is reasonably likely to produce clinical benefit in humans. In addition, human safety data in healthy volunteers is required to demonstrate the safety profile. As per the animal rule, the BAT development program consists of efficacy evaluation in two animal models and safety evaluation in healthy human volunteers. The efficacy evaluation against all seven serotypes was conducted in the Guinea pig (manuscript in preparation) model. Due to ethical constraints with the use of a large number of non-human primates, the efficacy is confirmed against one serotype in Rhesus macaques. The safety data was collected in human healthy volunteers. BAT received licensure by the United States Food and Drug Administration on 22 March 2013. It is currently the only licensed botulinum antitoxin against all known seven botulinum toxin serotypes and is approved for treatment of botulism in adults and pediatric patients.

The clinical profile of botulinum toxin, serotype A (BoNT/A) in Rhesus macaque is similar to those reported cases of human botulism [35, 36, 37]. Based on the similarities in pathogenesis and the ability to translate the efficacy data to humans, the Rhesus macaque is considered to be a relevant animal model for efficacy testing of BAT. A single botulinum toxin, serotype A was selected to evaluate the efficacy of BAT in Rhesus macaques. Initially, the disease was characterized in Rhesus macaque and then three [3] randomized, placebo-controlled studies were conducted to assess the efficacy of BAT.

Aerosol and oral deliveries of botulinum toxins are associated with high variability, and for oral exposure, the matrix in which the toxin is suspended can affect bioavailability [38]. For the purpose of these studies, the intravenous route of exposure was selected to achieve an accurate exposure dose. Safety evaluation of BAT had been conducted in normal healthy Rhesus macaques before initiation of efficacy studies. The efficacy studies were designed to test the null hypothesis that there would be a similar survival rate at the end of the study in the BAT and the placebo treated animals. The alternative hypothesis is that survival rate in BAT treated animals would be either higher or lower than the survival rate in the placebo group.

The efficacy was first assessed in a randomized, placebo-controlled study in Rhesus macaques in the prophylactic setting. To support the treatment indication, BAT was evaluated in a therapeutic setting where animals were treated at the onset of pre-defined clinical signs indicative of botulism. The results of these studies provided the evidence of effectiveness to support the licensure of BAT.

## Methods

### Animals, animal husbandry and veterinary care

All animal studies were performed at Lovelace Respiratory Research Institute (LRRI), Albuquerque, NM USA. A total of 130 Rhesus macaques (*Macaca mulatta*) weighed between 3kg to 7 kg (Global Research Supply, Sparks, NV) and were between 2 and 4.3 years of age. A higher sample size was used for the efficacy studies in order to achieve at least 80% power to detect a significant difference in survival rates between the treatment group and control group at alpha level of 5%. Monkeys were quarantined for 38 days and only clinically healthy monkeys were used in the study. Monkeys were individually housed in standard stainless-steel non-human primate cages conforming to the standards specified in the “Guide for the Care and Use of Laboratory Animals” [39]. All animals on the therapeutic efficacy studies were surgically implanted with an indwelling central venous catheter and fitted with a non-human primate jacket. The pre-established criteria for euthanasia included: paralysis; severe respiratory distress; prostrate and unresponsive to touch or external stimuli and loss of >25% of the body weight in order to prevent or alleviate pain and/or distress. The animals were scored for morbidity on a euthanasia scoring criterion (different than the clinical signs scoring) and a morbidity score of equal to or greater than 12 were considered for euthanasia. Animals were humanely euthanized and recorded as dead when they met the criteria for euthanasia.

All monkeys that met criteria for euthanasia or at the end of the study were sedated with ketamine (approximately 5mg/kg to 10mg/kg) by IM injections and euthanized using Euthasol (1.0-ml/4.5kg) intravenously.

### Ethics statement

Non-human primate research was conducted in compliance with the Animal Welfare Act and other federal statutes and regulations relating to animals and experiments involving animals and adhered to principles stated in the Guide for the Care and Use of Laboratory Animals [39]. All animal procedures were conducted under protocols approved by LRRI Institutional Animal Care and Use Committee. The LRRI is fully accredited by the Association for Assessment and Accreditation of Laboratory Animal Care International (AAALAC) and has an approved Office of Laboratory Animal Welfare Assurance (#A3083-01).

### Toxin

Partially purified *Clostridium botulinum* Neurotoxin Complex Serotype A (BoNT/A), produced at the University of Wisconsin, was used in all studies. The original stock of BoNT/A (Lot # A001195, Strain Hall) was diluted to working batch (batch 2a) and stored at -70C. The potency of the working batch of toxin was assessed using mouse potency assay every two years. The potency of the toxin batch was confirmed just before the study and the same batch of toxin was used for all of the studies described here. The potency of the serotype A toxin batch used was 172,559 mouse intraperitoneal lethal dose fifty (MIPLD_50_)/mL/25μg. Biosafety Level 2 practices were followed for the handling of BoNT/A.

### Immunization, plasmapheresis and BAT^®^ production

BAT is manufactured by Emergent BioSolutions (previously known as Cangene Corporation), Winnipeg, Manitoba, Canada. It is a hyperimmune plasma product prepared from horses that have been immunized with a specific serotype of botulinum toxoid or toxin. Horses were immunized with a specific serotype of botulinum toxoid and toxin to achieve high neutralizing antibody titers. Large quantities of plasma were collected using plasmapheresis. Following plasmapheresis, the immune globulin fraction was purified using a validated manufacturing process. This process for each antitoxin type includes cation-exchange chromatography to purify the immune globulin fraction, digestion with pepsin to produce F (ab') 2 plus F (ab') 2-related immune globulin fragments, anion exchange chromatography to remove the pepsin as well as other impurities and filtration. Removal of Fc using pepsin minimizes the potential for immunogenic reactions with equine products in humans. In addition, the manufacturing process includes two viral inactivation/removal steps; solvent/detergent (S/D) treatment and virus filtration.

The S/D treatment step is effective at inactivating known lipid-enveloped viruses such as equine encephalitis, equine arteritis, West Nile virus, equine infectious anemia, equine herpes virus, rabies, and equine influenza. The BAT manufacturing process also includes a robust filtration step that is effective in reducing the levels of some lipid-enveloped viruses (listed above) as well as non-enveloped viruses including equine rhinovirus, equine adenoviruses and equine parvovirus.

Following formulation, the individual botulism antitoxins are blended into the final heptavalent product. BAT lots used in these studies contained a total protein concentration of 56mg/mL (Lot # 2060401X) or 60 mg/mL (Lot #10805079). BAT lots used on the study contained an anti-serotype A titer of 10,399 units/vial (Lot #2060401X) or 8,496 (Lot #10805079) units/vial based on *in vivo* neutralization assay [40]. The potency and stability of BAT used in animal studies were confirmed. The intended clinical dose is 1 vial administered intravenously. The human dose was scaled to animal studies based on volume/kg basis (1x scaled human dose = 1 vial (volume) divided by 70 kg (average human body weight) and administered intravenously.

### Placebo

Botulism Antitoxin Placebo (normal equine immune globulin), is manufactured using a procedure similar to the manufacture of BAT described above. Placebo had a protein concentration of 50 mg/ml and potency of <0.257 Units/vial. The placebo was administered intravenously at a dose equivalent to protein dose (mg/kg) of the BAT product.

### Clinical observations/scoring

In the disease characterization study, animals were monitored for clinical signs hourly for the first 5 days following toxin exposure and every 4 hours thereafter until study termination at 14 days post-toxin exposure. Clinical signs observed are shown in Table 1. Animals in the PEP study were observed hourly after exposure until treatment at 4 hours post-exposure and continued until day 5 post-exposure. The frequency of observations shifted to every 4 hours beginning on day 5 and continued until study termination at 14 days post- exposure. Animals in the therapeutic studies were observed up to 3 pre-toxin exposure observations for baseline clinical severity scores (days -2, -1 and 0). Following toxin exposure, clinical severity scores were recorded every hour beginning at 24 hours post exposure and up to study day 5. Beginning on day 5, the frequency was shifted to every 4 hours until study termination.

**Table 1:** Clinical signs and associated clinical severity scores used in all studies.

Clinical Signs	Observation	Clinical Severity Score Assigned
**Ptosis**	**Absent**	**0**
	**Slight**	**1**
	**Marked**	**2**
**Muscular Weakness**	**Normal**	**0**
	**Hunched Posture**	**1**
	**Prostrate but able to rise**	**2**
	**Prostrate unable to rise**	**3**
**Respiratory Distress**	**Normal breathing**	**0**
	**Open mouth shallow (Thoracic)**	**1**
	**Gasping and jerky (Abdominal)**	**3**
**Oral Discharge**	**Absent**	**0**
	**Present**	**1**
**Nasal Discharge**	**Absent**	**0**
	**Present**	**1**

### Supportive care

Supportive care was administered to all of the intoxicated animals in both of the therapeutic efficacy studies.

#### Parenteral nutritional support

Monkeys received parenteral nutritional support via the central venous catheter immediately following treatment with either BAT or placebo. An ambIT^®^ infusion pump (Summit Medical Products, Inc., Salt Lake City, Utah) was used for delivery of parenteral nutrition. A parenteral nutritional solution formulated with 15% protein, 30% lipids, and 55% carbohydrates prepared by a local pharmacy were used. Multivitamins were also added to the nutritional solution daily as recommended [41]. The amount of nutritional solution administered was estimated on the basis of resting energy rate (RER, 70*kg ^0.75^ (kcal/day) [42] as determined by individual monkey body weight, and the concentration (kcal/mL) of the support used. The volume of parenteral nutrition administered was changed depending on the clinical condition of an animal.

#### Fluid support

Hydration status was subjectively assessed via a modified grading system [43]. Briefly, a dehydration score of 5–6% for a subtle loss of skin elasticity, 6–8% for moderate loss of skin elasticity, 10–12% when there is severe loss of skin elasticity (tented skin stands in place); sunken eyes in orbits and possible signs of shock, 12–15% when there are definite signs of shock. Intravenous fluid therapy with lactated Ringer’s solution (LRS; Hospira, Inc., Lake Forest, Ill) was administered via the Broviac catheter using the ambulatory infusion pump after administration of parenteral nutrition. The volume of LRS required was determined by calculating the sum of the hydration deficit and the daily maintenance fluid requirement, using the following calculations:

1. Hydration deficit (L) = body weight (kg) * % dehydration as a decimal * X%, where X = the percentage of the hydration deficit to be replaced during a given period.
2. Daily maintenance fluid requirement (L) = (70*kg^0.75^ = 70 is metabolic body size, body weight, and the exponent 0.75 is a scaling parameter for metabolic body mass).

Replacement fluids were administered slowly to allow adequate time for equilibration of fluids and electrolytes and to avoid potential complications with the volume overload.

### Design of studies

A study was conducted to establish LD_50_, define the clinical markers of symptomatic disease and identify the optimal triggers for therapeutic intervention studies. Because the animal efficacy studies provide the primary evidence of effectiveness for licensure, they were designed and performed with the approach used for human clinical trials. All of the studies described herein were conducted in accordance with Good Laboratory Practice (GLP) regulations.

#### Disease characterization study

The LD_50_ for BoNT/A in monkeys had previously been reported [35]. To confirm the lethal dose for the current preparations of BoNT/A in monkeys an LD_50_ study was conducted. A detailed clinical course and clinical markers were also assessed. Four groups of monkeys (two /gender/group) were assigned to each toxin dose group using a stratified body weight randomization procedure and were given 160, 60, 40, or 25 MIPLD_50_/kg of Botulinum Neurotoxin Type A by intravenous injection. To minimize bias, the observers were blinded to the doses for which the animals were exposed. The typical clinical signs of botulism observed and the clinical scoring assigned for each clinical sign are shown in Table 1. The endpoint analysis included mortality and the median time to onset of clinical signs and death.

#### Post-exposure prophylaxis study

This PEP study was a randomized and placebo-controlled conducted according to GLP. Thirty (30) monkeys were randomly assigned to three groups of ten (5animals/gender) and exposed intravenously to 4x monkey LD_50_ of BoNT/A (104 MIPLD_50_/kg). Based on the disease course identified in the characterization study, approximately 4 hours following intoxication with ~4x LD_50_/kg of BoNT/A (104 MIPLD_50_/kg) and before onset of any of the clinical signs, each group received either a single intravenous dose of placebo or BAT (Lot # 2060401X) at either 1x scaled human dose (0.16 mL/kg or 149U/kg of anti-serotype A) or 0.1x scaled human dose (14.9 U/kg) intravenously. Animals were monitored frequently for clinical signs and mortality. The primary efficacy endpoint was survival and the secondary endpoint was median time-to-death, defined as time between intoxication (on day 0) and death during the 14-day study period. In addition, the incidence of clinical signs of intoxication were also evaluated and compared between treatment and placebo groups.

#### Therapeutic efficacy studies

A randomized, controlled and blinded pilot study (Study 1) was conducted under GLP to evaluate the therapeutic efficacy of BAT and to support the pivotal licensure study. The objective of this pilot study was to determine whether administration of BAT at the onset of clinical signs in combination with minimal nutritional support decreases the progression of clinical signs and occurrence of death in monkeys prior to setting up a pivotal licensure study. Eighteen (18) monkeys were randomly assigned to two groups (8-10/group) and exposed to a 1.7x LD_50_/kg of BoNT/A on day 0. At the onset of first clinical sign of botulism (ptosis, muscular weakness, respiratory distress), animals were dosed intravenously with either BAT at 1x scaled human dose (121 U/kg of anti-serotype A, Lot# 10805079) or equivalent protein dose of placebo. Intravenous nutritional support was initiated to all intoxicated animals immediately following treatment. On day five, the frequency of clinical observations was reduced to every four hours till the end of the study. The primary efficacy endpoint was survival, defined as the percentage of intoxicated monkeys that survived to day 14. The secondary endpoint was median time-to-death. Additionally, the incidence of clinical signs and recovery from clinical signs of intoxication were also evaluated and compared between groups. Following the preliminary study, the pivotal efficacy study (Study 2) supporting licensure was conducted.

The pivotal efficacy study was a randomized; placebo-controlled, and blinded study conducted under GLP regulation. Sample size estimation for the study was based on the survival results from the pilot study. A total of 30 monkeys per group were necessary to achieve at least 80% power to detect a 45% difference in survival between the groups at alpha level of 5%. Animals were exposed to ~1.7x LD_50_/kg dose of BoNT/A on day 0 and were monitored regularly as described above. Monkeys were treated individually with either BAT at 1x scaled human dose (121U/kg of anti-serotype A, Lot # 10805079) or placebo at the onset of clinical disease and nutritional support was initiated immediately after treatment. Clinical observations and scoring were continued after treatment. The sum of severity scores at each time point was calculated to give an animal’s total clinical score.

The primary efficacy endpoint was survival, defined as the percentage of intoxicated monkeys that survived to day 21. The secondary efficacy endpoints included median time to death (MTD), time to onset of (severe) clinical signs and time from onset of clinical signs to recovery. The duration of each clinical sign was also summarized by treatment group. The average severity score over time was compared between treatment groups.

### Statistical analysis

The median lethal dose and 95% Confidence Interval (CI) were estimated using a binary logistic regression model. Median times to clinical signs of intoxication and median times to death were calculated with 95% CI using Kaplan-Meier statistics. The survival rates for each dose level of BAT treatment were compared to the placebo group using the two-tailed Fisher’s exact test. An overall significance level of 5% was used and the probability of a type 1 error for each test was adjusted for multiple comparisons. Kaplan-Meier curves along with log-rank tests were used to compare the MTD between groups.

For the therapeutic study, the survival rates were compared using a two-tailed Fisher’s exact test, with significance set at α = 0.05. As part of the secondary analysis, median time to development of clinical signs, MTD from intoxication, and median time to recovery were calculated using the Kaplan-Meier statistics. The MTD were compared between groups using log-rank tests.

Treatment effect on severity scores over time was analysed using a linear mixed model fitted to the sum of severity scores (excluding food intake or including food intake over time). The fitted model included fixed effects for treatment, time and treatment by time interaction with significance set at alpha of 0.05.

## Results

### Disease characterization study

In intoxicated monkeys, there was a rapid clinical course with a short interval between the onset of clinical signs and death. The median lethal dose was estimated to be 26 MIPLD_50_/kg (95% CI: 24–28 MIPLD_50_/kg) and all animals that received 160, 60, and 40 MIPLD_50_/kg of body weight died or were euthanized. The MTD was directly related to the toxin dose (Table 2).

**Table 2:** Median time to onset of clinical signs (in hours) and 95% confidence interval for each clinical sign across toxin dose groups, and median time to death (in hours) and range.

*Clinical sign*	*Intravenous Toxin Dose* *Median clinical onset time in hours (Range)*			
	25 MIPLD_50_/kg (n = 4)	40 MIPLD_50_/kg (n = 4)	60 MIPLD_50_/kg (n = 4)	160 MIPLD_50_/kg (n = 4)
**Ptosis**	42 (40, 61)	30 (19, 41)	21.5 (17, 32)	17.5 (17, 19)
**Muscular Weakness**	51 (40, 62)	39.5 (19, 41)	30.5 (26, 36)	18.5 (16, 20)
**Respiratory Distress**	64.5 (62, 89)	39.5 (36, 43)	34.5 (35, 36)	19 (18, 23)
**Oral Discharge**	-- (102, --)	47 (34, --)	36 (35, 41)	-- (20, --)
**Nasal Discharge**	--	--	-- (33, --)	-- (20, --)
Death*	--	47 (40, 105)	36.5 (35, 43)	23 (20, 24)

-- Not calculable due to limited number of events (i.e. limited observations of clinical sign and/or death).

* Only median time to death and range was reported

Clinical signs of botulism followed a general pattern of descending paralysis and were comparable to previously established findings in this model. In general, ptosis was usually the first observed clinical sign with the highest incidence (observed in >90%) and was observed in conjunction with muscular weakness and/or respiratory distress. The MTD in the highest dose group was 23 hours (range: 20–24 hours), which occurred within a short time after the onset of the first clinical sign (ptosis, 17.5 hours), indicating a rapid progression of the disease. The progression from ptosis to muscular weakness and respiratory distress with 60 and 40 MIPLD_50_/kg was similar to those given 160 MIPLD_50_/kg, except it was much more rapid. The onset of any of these clinical signs alone or in combination was determined to be a trigger for initiation of treatment in the therapeutic efficacy studies. Based on clinical progression, it was clear that a dose of ~ 2x monkey LD_50_/kg (~50MIPLD_50_/kg) would not provide an appropriate window for treatment as the MTD from the median time of ptosis was only 15 hours. Thus, a slightly lower dose of 1.7xmonkey LD_50_ toxin/kg (~ 40MIPLD_50_/kg) which resulted in 100% mortality and a wider range for time to death was selected for use in the therapeutic intervention studies.

### Post-exposure prophylaxis study

**Fig 1:**
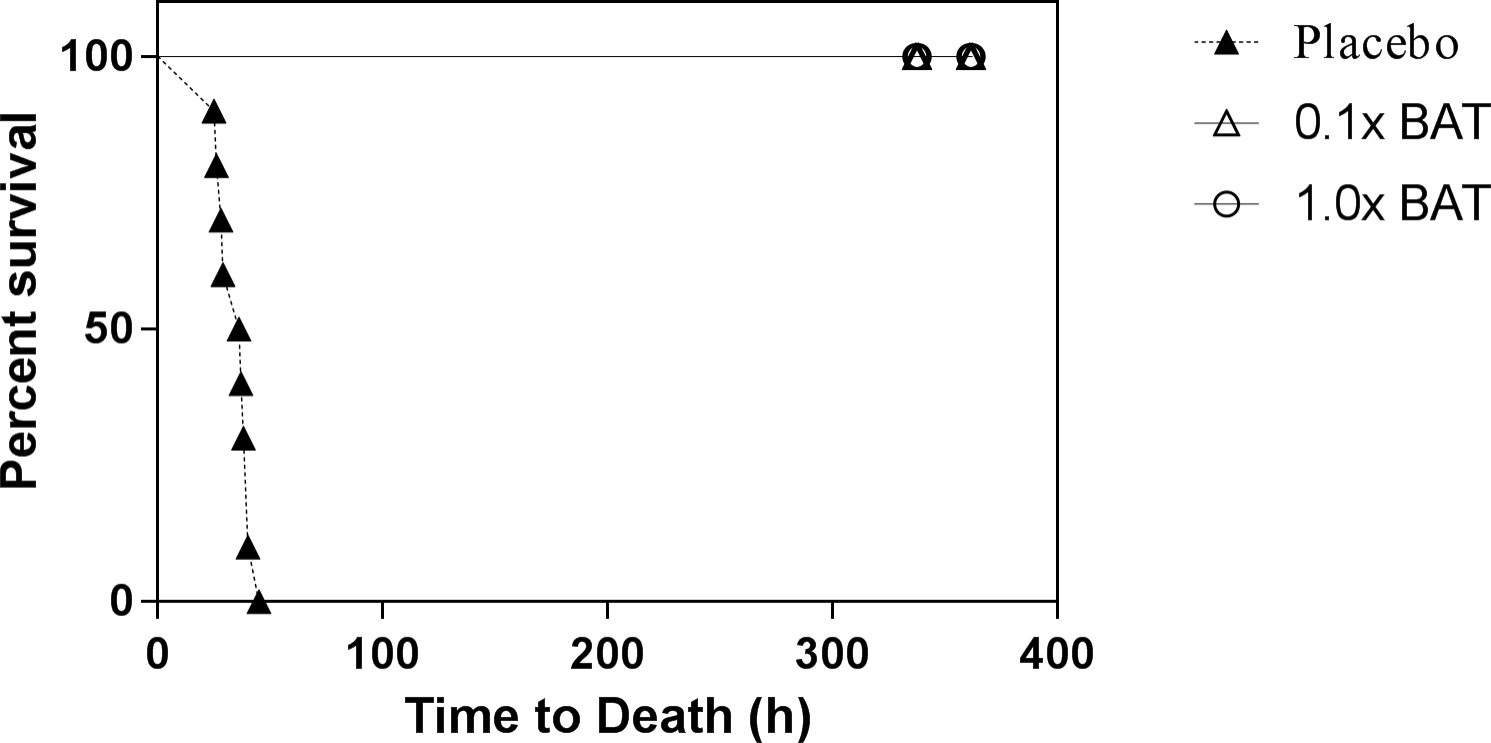
Kaplan-Meier survival curves for BoNT/A intoxicated monkeys treated with BAT. Monkeys were intoxicated with 4x LD_50_/kg dose of BoNT/A and treated intravenously with either dose of BAT (1x or 0.1x scaled human dose) or placebo after 4 hours post-intoxication before the onset of clinical signs. The proportion of animals that survive to 14 days post-toxin exposure in is shown for each group.

As a measure of efficacy, BAT was evaluated in a post-exposure prophylaxis setting. All animals administered BAT (both 1x and 0.1x scaled human dose groups) survived until the end of the study (day 14) indicating complete protection from lethal effects of BoNT/A (Fig 1). In contrast, all placebo treated animals died (Table 3). This survival rate in the treated group compared to placebo was statistically significant (p<0.001, Fisher’s exact test). Animals were monitored very closely and no safety related findings were observed in animals treated with BAT.

**Table 3:** Summary of mortality and median time to death data-PEP study.

*Groups*	*Number Survived/Number on the Study*	*Fisher Exact Test p-Value*^*a*^	*Median Time to death in Hours (95% Confidence Interval)* *	*Log-Rank Test p-Value*
BAT, 1x Dose	10/10	<0.001	>362 (.,.)	<0.001
Bat, 0.1xDose	10/10	<0.001	>362 (.,.)	<0.001
Placebo	0/10	-	36.5 (28.0. 39.0)	-

^a^ Adjusted for multiple comparisons

* Confidence Intervals (CI) are presented except when the estimated survival distribution of the group did not cross 0.50, in which case they are shown as (., .).

The MTD for the placebo group was 36.5 hours (95% CI: 28.0, 39.0, Table 3) and was significantly shorter compared to BAT treated group (>362 hours). The incidence of clinical signs of toxicity for all groups is given in Table 4. Except one animal in the group treated with 0.1x BAT dose, all animals showed no clinical signs indicating the efficacy of BAT in preventing the lethal effects botulinum toxin (Table 4.). The majority of animals treated with placebo showed clinical signs associated with botulism. The median time to onset of clinical signs in the placebo group is given in Table 5. The signs of botulism intoxication in the placebo appeared at about 27.5 hours (median time to ptosis, Table 5) and death occurred within a short period after the first observation of clinical signs demonstrating a rapid progression of the disease in the absence of antitoxin treatment.

**Table 4:** Incidence of clinical signs in BAT and placebo group-PEP study.

Clinical Observational Endpoint	BAT (1xdose) *(*n = 10)	BAT (0.1x dose) (n = 10)	Placebo (n = 10)
Ptosis, n (%)	0 (0%)	0 (0%)	10 (100%)
Muscular weakness, n (%)	0 (0%)	0 (0%)	10 (100%)
Respiratory Distress, n (%)	0 (0%)	0 (0%)	9 (90%)
Oral discharge, n (%)	0 (0%)	1 (10%)	7 (70%)
Nasal discharge, n (%)	0 (0%)	0 (0%)	4 (40%)

**Table 5:** Median time to onset of clinical signs in placebo group-PEP study.

*Endpoint*	*Median time to onset of clinical signs (hours)*	*95% CI on median time to onset of clinical signs*
Ptosis	27.5	(23, 29)
Muscular Weakness	28.5	(23, 29)
Respiratory Distress	30	(29, 31)
Oral Discharge	37	(27, 38)
Nasal Discharge	34	(32, --)

-- Not calculable due to limited number of events.

### Therapeutic efficacy

In order to determine the therapeutic efficacy of BAT, a small pilot efficacy study (Study 1) and a large pivotal therapeutic (Study 2) efficacy study were conducted in monkeys. Both were randomized; placebo controlled and blinded GLP studies.

In Study 1, animals were exposed to a 1.7xLD_50_/kg dose of BoNT/A and treated individually with the 1x scaled human dose of BAT (121 U/kg of anti-serotype A toxin) at the onset of the first definitive clinical sign(s) of botulism. Supportive care was initiated in all animals immediately after the treatment. Supplemental fluid support was also provided to dehydrated animals to mimic the expected clinical care. Clinical signs observed in all animals following intoxication were consistent with botulinum intoxication (Table 6). At the onset of any of the designated clinical signs, treatment was initiated. The median time to development of initial clinical signs was comparable for both treatment and placebo groups, suggesting that there was no bias between the groups. Additionally, as expected, the onset times were delayed due to the lower dose (1.7x LD_50_/kg) used for intoxication compared to the post-exposure prophylaxis study (Table 5).

**Table 6:** Median time to onset of clinical signs in monkeys-Therapeutic Study 1.

*Clinical sign*	*Median Time to Onset in Hours(95% Confidence Intervals)*	
	Placebo	BAT
**Ptosis**	51 (39, 59)	42.5 (37, 60)
**Muscular Weakness**	47 (41, 50)	45.5 (38, 48)
**Respiratory Distress**	56 (51, 63)	56.0 (37, 65)
**Oral Discharge**	60 (47, 64)	61.5 (47, 77)
**Nasal Discharge**	90 (--, --)	-- (44, --)

-- Kaplan-Meier median or confidence interval estimates could not be estimated due to limited number of events.

The MTD and survival is shown in Table 7. Five of 10 animals from the BAT treatment group recovered completely and survived to the end of the study with no botulism related clinical signs. The remaining animals from treatment group and all animals from the placebo group were euthanized.

**Table 7:** Survival and median time to death for monkeys treated with BAT at the onset of systemic disease-Therapeutic Study 1.

*Treatment*	*Survival (%)*	*Fisher’s Exact Test (p-value)*	*Median Time to Death in Hours (95% CI)*	*Log-rank Test (p-value)*
**BAT**	5/10 (50%)	p = 0.044	-- (54, --)	p = 0.003
**Placebo control**	0/7(0%)		65 (55, 74)	

-- The Kaplan-Meier median and the upper confidence bound were not calculable due to ≥ 50%censoring included.

The primary efficacy (survival at Day 14) endpoint was positive in Study 1 with significantly enhanced survival (50%) with 1x scaled human dose of BAT compared to 0% survival in the placebo group (p = 0.044, Fisher’s exact test). The median survival time was significantly prolonged in the group that received BAT versus placebo (p = 0.003, log-rank test, Table 7). The time from onset of clinical signs to recovery for the BAT treated group was 108 hours (95% CI: 49, 194). All seven intoxicated placebo control animals were euthanized prior to the study end and the MTD from the onset of clinical signs for the placebo group was 17 hours (95% CI: 10, 23) indicating a rapid progression of the disease in these animals in the absence of antitoxin treatment.

All the study animals tolerated receiving supportive care. Once voluntary food and water consumption was observed, the administration of nutritional support was gradually decreased. Total mean volume administered to BAT and placebo group was 357.3 ± 220.4 and 95.6 ± 54.1 mL, respectively, indicating longer nutritional support for BAT treated groups. Additionally, only 3 out of 10 animals from the BAT group were treated with fluid support for dehydration compared to none in the placebo group. The minimal nutritional and fluid support requirement by the placebo group is due to the fact that most animals in this group were dead or euthanized by day 4.

### Therapeutic efficacy—study 2

Due to the large sample size (n = 30/treatment group) and requirement of nutritional support for each animal, the study was conducted in three cohorts. Similar to Study 1, animals from the pivotal licensure study were exposed to 1.7xLD_50_/kg dose of BoNT/A and treated individually with 1x scaled human dose of BAT (121 U/kg of anti-serotype A toxin, Lot#10805079) at the onset of clinical signs. Clinical signs in all animals were comparable to the signs observed in Study 1 and consistent with botulinum intoxication. The time to onset of initial clinical signs (trigger) for initiation of treatment was comparable between BAT and placebo control groups, indicating absence of bias in group assignment/treatment (Table 8).

**Table 8:** Estimated median time to onset of clinical signs for monkeys intoxicated with 1.7xLD_50_/kg of BONT/A-Therapeutic Study 2.

Clinical sings	Median Time to Onset in Hours (95% Confidence Intervals		Log-Rank Test (p-value)
	Placebo Control	BAT	
**Ptosis**	64 (55, 67)	62 (55, 66)	p = 0.294
**Muscular weakness**	59 (52, 63)	60.5 (53, 63)	p = 0.129
**Respiratory distress**	58 (53, 63)	59.5 (55, 63)	p = 0.347
**Oral discharge**	56 (53, 60)	61.5 (58, 65)	p = 0.072
**Nasal discharge**	84 (67, 110)	107 (90,–) ^a^	p = 0.048^b^

^a^ The upper bound of the confidence interval (CI) could not be estimated due to the limited number of events.

^b^A statistically significant (α = 0.05) difference was detected using the log-rank test.

**Fig 2:**
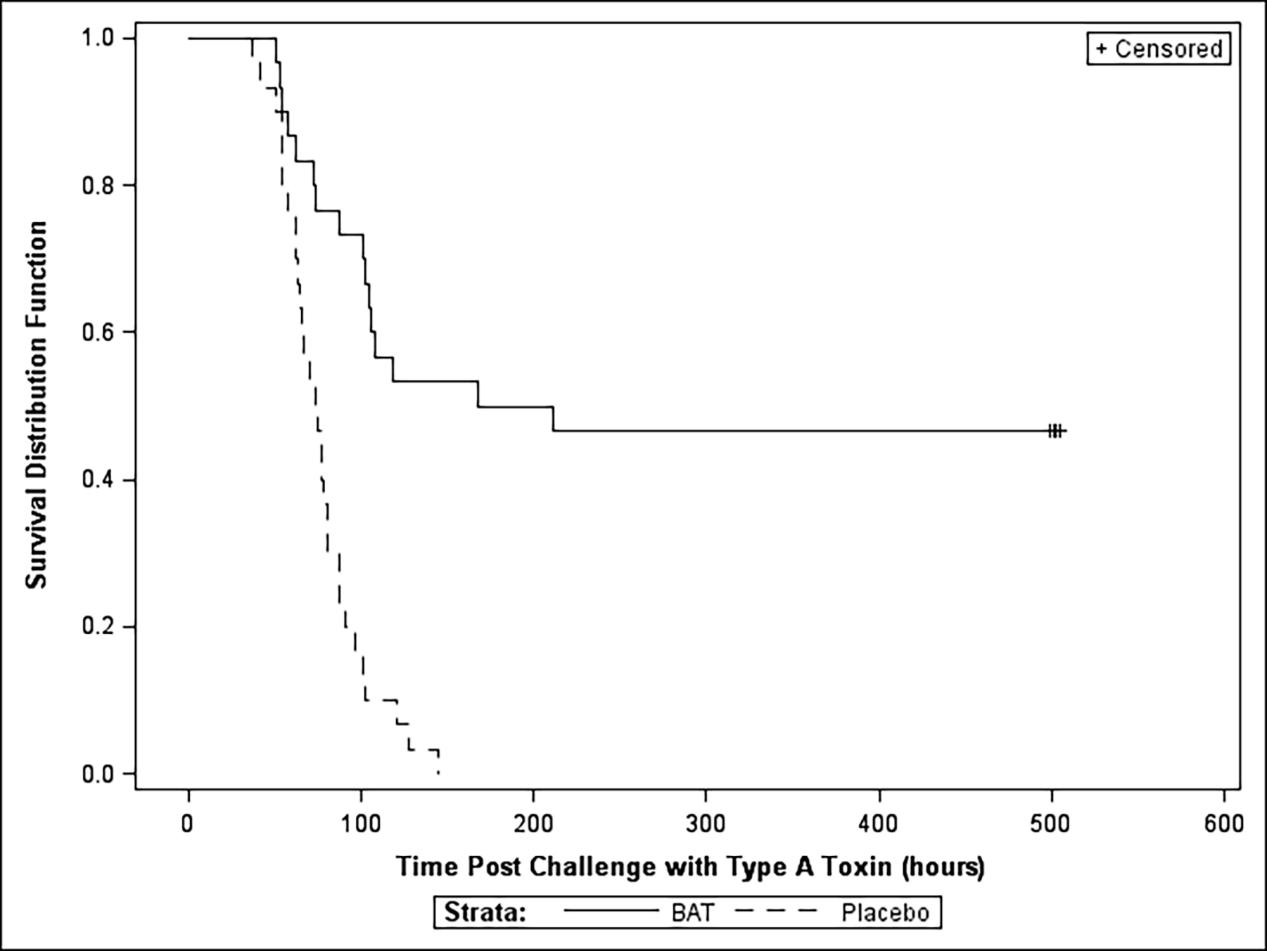
Kaplan-Meier survival curves for BoNT/A intoxicated monkeys treated with BAT. Groups of monkeys (n = 30/group) were intoxicated with 1.7x LD_50_/kg dose of BoNT/A and monitored hourly for the onset of clinical signs. Monkeys were individually administered with either BAT at 1x scaled human dose or placebo intravenously after the onset of clinical signs indicative of botulism (Study 2) and monitored for survival to 21 days post-toxin exposure.

The survival rate and the MTD are given in Table 9 and a Kaplan-Meier survival curve is shown in Fig 2. The primary efficacy endpoint was achieved with 46.7% (14 of 30) survival in the BAT treated group compared to 0% (0/30) survival in the placebo group. This difference in survival between the two groups was statistically significant (p<0.0001, Fisher’s exact test). The MTD was significantly increased (p < 0.0001, log-rank test) in the BAT (median, 189.5 hours) treated group compared to the placebo group (median, 74.5 hours days).

**Table 9:** Survival and median time to death for monkeys treated with BAT at the onset of systemic disease-Therapeutic Study 2.

Treatments	Survival rate (No. of Survivors/No. in Group%)	95% Confidence Interval	Fischer’s exact test (p-value)^a^	Kaplan Meier median Time to Death in Hours (95% Confidence Intervals)	Log-Rank Test (p-value)^c^
BAT	0.47 (14/30)	(0.28, 0.66)	<0.0001	189.5 (102, --)^b^	<0.0001
Placebo Control	0.00 (0/30)	(0.00, 0.12)		74.5 (63,81)	

^a^ A statistically significant (α = 0.05) difference was detected using Fisher’s Exact test.

^b^ The upper bound of the confidence interval could not be estimated due to the limited number of events (i.e. 14 animals survived until study termination).

^C^A statistically significant (α = 0.05) difference was detected using the log-rank test.

Duration of clinical signs (disease) was affected by the treatment with BAT. For example, the BAT treated animals typically had milder signs that persisted for longer duration, while, in contrast, the animals in the placebo group progressed to subsequent severe signs and eventual death more rapidly (Table 10). It is also important to note that nearly half of the animals in the BAT treated group survived to the end of the study. This extension to the duration of clinical signs is indicative of a treatment effect of BAT in halting the progression of the disease among treated animals.

**Table 10:** Duration of clinical signs in monkeys treated with BAT-Therapeutic Study 2.

*Clinical Sign*	*Number of Animals showing the sign/Total Number Animals Mean (SD) Duration*	*BAT (Hours)*	*Placebo (Hours)*
**Ptosis**	N	23/30	26/30
	Mean (SD)	27.5 (22.5)	21.3 (23.6)
**Muscular weakness**	N	27/30	28/30
	Mean (SD)	69.6 (65.3)	21.5 (16.6)
**Respiratory distress**	N	27/30	28/30
	Mean (SD)	63.6 (69.0)	17.1 (14.3)
**Oral discharge**	N	26/30	27/30
	Mean (SD)	55.2 (48.4)	22.1 (17.8)
**Nasal discharge**	N	14/30	13/30
	Mean (SD)	66.9 (77.9)	18.5 (23.9)

Time from onset of clinical signs to recovery (time when sum of clinical scored returned to zero) was also treatment dependant. A median time to recovery of 137 hours (95% CI: 118, 222) was observed for animals treated with BAT. Since no animals in the placebo group recovered, the median time to recovery estimation was not possible.

The administration of nutritional support was gradually decreased once voluntary food and water consumption was restored due to recovery. Similar to Study 1, the mean volumes administered to BAT and placebo group were 328.3±264.4 and 115.9±79.1mL, respectively, indicating longer nutritional support for BAT treated animals. Similarly, 19 out of 30 animals from the BAT groups were treated with fluid support for dehydration compared to 6/30 in the placebo group.

The mean volume received by BAT and placebo were 525.9± 402.3 mL and 222.6±168.1mL, respectively. Similar to the previous study, the increased nutritional and fluid support is due to higher survival rate and longer survival time observed for treatment group. In the placebo group the nutritional support alone was not able to rescue these animals from the toxin effects.

**Fig 3:**
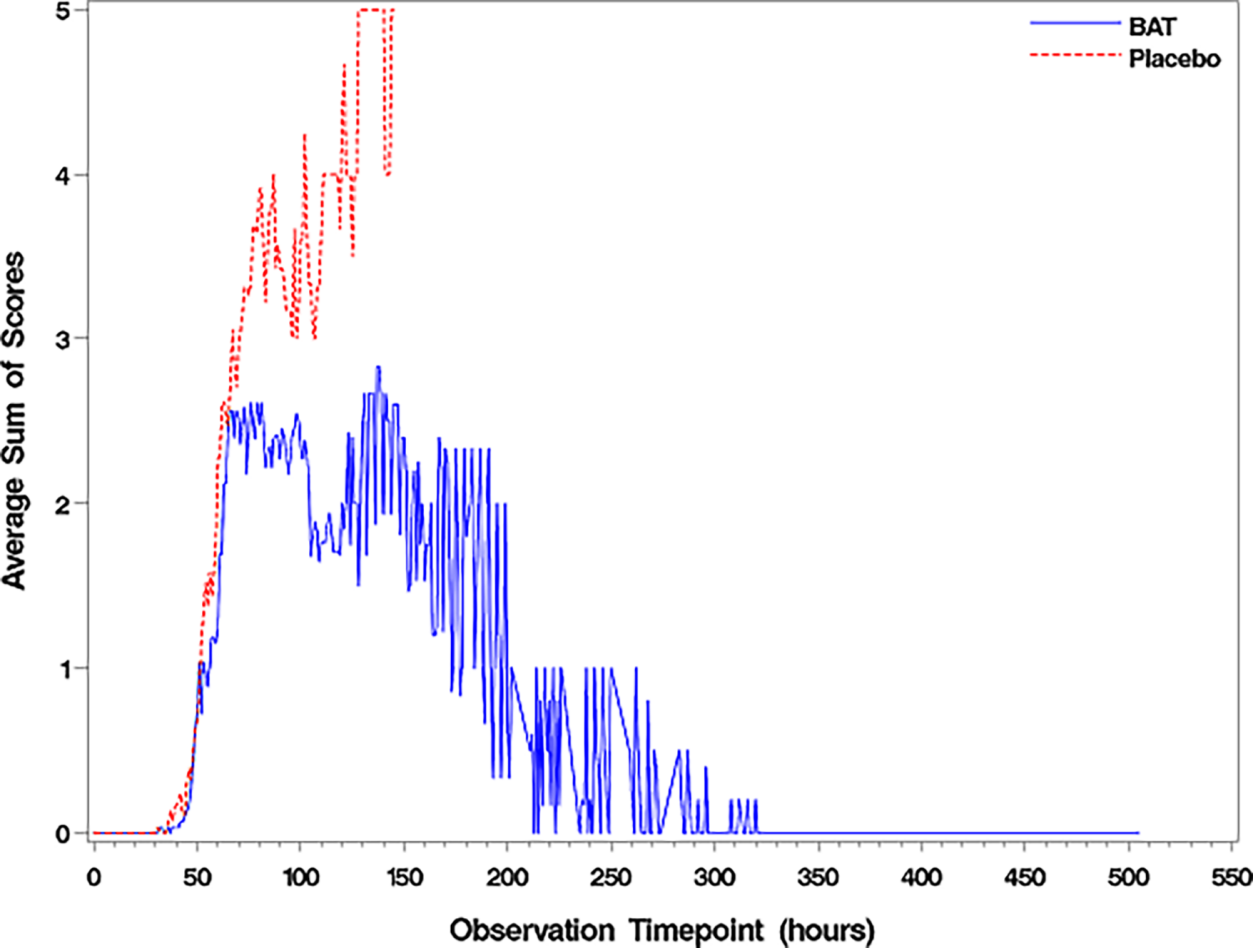
Clinical severity scores were calculated for each individual animal at a given observation time point. Monkeys were intoxicated with 1.7x LD_50_/kg dose of BoNT/A and treated intravenously with either BAT at 1x scaled human dose or placebo after the onset of clinical signs indicative of botulism. Clinical signs were monitored during the course of infection and the clinical severity scores were calculated for each individual animal at given observation time point after intoxication. The designation for the clinical score for each sign is shown in Table 1. The data reflects the average sum of severity scores over time for both BAT (1x scaled human dose) and the placebo group.

The clinical severity scores for each sign was calculated for each individual animal at a given observation time point in Study 2. The averaged sum of severity scores at each observation point by treatment group was generated to demonstrate the treatment effect and recovery (Fig 3). At the onset of clinical signs, the clinical severity score was comparable between groups. However, the clinical severity score for the placebo group increased over time until all animals were dead. The severity score decreased over time for survivors until ~300 hours post-intoxication, when all the survivors became asymptomatic and recovered with no clinical score. There was a statistically significant difference in the clinical score data between treatment and placebo groups with the significance level of alpha = 0.05 for both the total sum of severity scores (including food intake) and the average sum of severity scores (excluding food intake, Fig 3). All the non-survivors in the BAT group were dead by 169 hours post-intoxication. The median clinical severity score for non–survivors of BAT and placebo group were 6.5 (range, 3 and 9) and 8 (range, 3 and 11), respectively.

## Discussion

BAT is the equine derived heptavalent antitoxin for treatment of symptomatic botulism caused by documented or suspected exposure to BoNT serotypes A, B, C, D, E, F, and G in adult and pediatric patients which was licensed under the “Animal Rule” by the FDA based on the demonstrated efficacy conducted in animal models of botulism and demonstrated safety in animal and humans. The animal model data from one species which formed the part of the evidence of efficacy under this rule are described in this report. In addition to animal efficacy data, the safety of BAT was demonstrated in animals and healthy human volunteers.

The toxin exposure study in monkeys resulted in clinical signs that were directly comparable to human botulism, confirming the suitability of the model for demonstrating the efficacy of BAT treatment as per the requirements of the “Animal Rule”. The time to onset in human botulism following exposure to BoNT is highly variable and depends on serotype [29], dose [44], and route of exposure [45, 46, 47, 48, 49, 50]. Consistent with previous findings [35], the present study results indicated an inverse relationship between the doses of BoNT/A and the survival times. Our disease characterization data indicated that the post-exposure therapeutic window is short even at lower toxin dose levels due to the acute nature of the disease; therefore, to be effective in therapeutic intervention, it was clear that the antitoxin must be given at the earliest onset of clinical disease.

In the PEP study, a single dose of BAT resulted in 100% survival after a lethal intravenous exposure to BoNT/A and prevented the onset of disease as evidenced by the absence of clinical signs. Previously, it has been shown that pre-exposure prophylactic treatment with the equine antitoxin is needed to completely prevent lethality in monkeys following inhalational exposure [51]. To our knowledge, the current study is the first in which monkeys were completely protected from lethal effects of toxin given intravenously with antitoxin treatment given 4 hours after exposure to lethal doses of toxin. The data from the PEP study suggest that the toxin is not internalized in lethal doses to nerve terminals and circulating toxin is available for neutralization for at least up to 4 hours post-exposure in this model. These findings are consistent with available data in mice and rats suggesting that the toxin without biotransformation is held in the plasma compartment even after initial re-distribution [52, 53]. Although intravenous exposure route used in these studies is not relevant for human exposure, it reflects the extreme route of exposure and the window for treatment could be even longer in humans due to delay in absorption of toxin via gut epithelium.

When given after the onset of the disease, BAT significantly increased the overall survival and MTD in animals with clinical evidence of systemic botulism disease. This therapeutic efficacy was reproducible in the subsequent pivotal GLP efficacy study. In contrast to a much smaller window of opportunity (about 10 min) for protection against systemic intoxication observed using monoclonal antibodies in mice model [53], the window for treatment with antitoxin in this model was two to two and a half days based on the time to onset of clinical signs in the therapeutic studies. The smaller window in mice model could be due to differences in the toxin preparation and the dose used. The survival rate (~46.6%) observed in intoxicated animals treated with BAT was statistically significant but still lower than survival rates reported clinically (i.e. 85–90%) for foodborne botulism following antitoxin treatment. Conversely, the survival rate of placebo treated animals (0%) is lower than that observed clinically among patients not receiving antitoxin (i.e. 46%) treatment [54]. This difference observed in humans is likely due to the additional supportive care (respiratory support) available in hospital setting when required in clinical practice. It is anticipated that the window of opportunity for treatment of foodborne botulism is much wider in humans than in monkeys exposed intravenously due to the time required for absorption from the gastrointestinal tract. Due to the systemic route of exposure and the lethal amount of toxin administered (i.e. close to 2x LD_50_), the animal model is robust and represents the worst-case scenario.

The antitoxins for BoNTs work by clearing toxin in circulation and inhibiting the binding of the toxin to the neuronal cell surface receptor [53,55]. Therefore, it has been suggested that the treatment with an antitoxin has limited value in symptomatic patients as most toxin will have already been internalized and be protected in the intracellular environment. The clinical experience with both foodborne botulism and wound botulism have clearly suggested the association between early administration of antitoxin and improved survival, length of hospital stay and use of ventilation [54,55,56,57,58,59]. Our results support this correlation as there was a significant difference in the survival rates observed between post exposure prophylactic and therapeutic efficacy studies. However, this report provides data to show a statistically significant survival among monkeys with evidence of systemic disease and suggests a wider window of opportunity for therapeutic antitoxin.

Clinical severity scores collected in the presence of supportive care are relevant for assessing the predictive efficacy of BAT in human patients because of their comparability to the clinical scenario. Treatment with BAT reduced the severity and delayed the progression of the disease, which is consistent with the known mechanism of action of the antitoxin. Although intravenous administration of BAT resulted in an immediate distribution within the circulatory system, the severity scores of treated animals increased at a rate comparable to controls until ~72 hours post-intoxication. These findings are consistent with the clinical experience, where administration of antitoxin did not cause an immediate cessation in the clinical progression but did minimize the subsequent severity of the disease [1]. Only a small number (<10%) of BAT treated animals died or were euthanized due to the moribund state resulting from toxin effects after day 5 post intoxication. These deaths could have been avoided in a comparable clinical setting where more aggressive support measures such as respiratory care are available. The duration of the recovery phase in human cases can range from several days to many months depending on the severity of the disease, serotype involved [60, 61, 62] and time of treatment [59]. In contrast, the monkeys in the treatment group that displayed severe muscular weakness and ptosis did recover completely within 21 days with no residual clinical signs of botulism. A comparatively rapid recovery was also observed in infants with botulism with just supportive care alone [63] or with antitoxin treatments [64].

In conclusion, a single humanized dose of BAT administered to symptomatic monkeys following intravenous exposure to BoNT/A resulted in statistically significant survival benefit and improved clinical signs compared to placebo controls. This statistically significant enhancement in survival and reduction in severity of clinical signs of intoxication is expected to translate into a clinical benefit. The results of these studies along with the efficacy data against all seven serotypes in guinea pigs (data not shown) provided the evidence of effectiveness for the licensure of BAT under the “Animal Rule” in the US.

**Trade marks**: Emergent BioSolutions, Protected by Emergent BioSolutions™, Cangene, BAT®, and any and all Emergent BioSolutions Inc. brand, product, service and feature names, logos and slogans are trademarks or registered trademarks of Emergent BioSolutions Inc. or its subsidiaries in the United States or other countries. All rights reserved. BabyBIG® is a trademark of California Department of Health Services. ambIT® is a trademark of Ambit Biosciences Corporation. All other brand, product, service and feature names or trademarks are the property of their respective owners.
